# Sodium-Glucose Cotransporter-2 (SGLT-2) Inhibitors: Benefits in Diabetics With Cardiovascular Disease

**DOI:** 10.7759/cureus.10783

**Published:** 2020-10-03

**Authors:** Saba Aftab, Rishwanth Vetrivel Suresh, Nazleen Sherali, Muhammad Daniyal, Nicholas Tsouklidis

**Affiliations:** 1 Medicine, Hamdard College of Medicine and Dentistry, Karachi, PAK; 2 Medicine, California Institute of Behavioral Neurosciences & Psychology, Fairfield, USA; 3 Health Care Administration, University of Cincinnati Health, Cincinnati, USA; 4 Medicine, Atlantic University School of Medicine, Gros Islet, LCA

**Keywords:** sodium-glucose cotransporter-2, dapagliflozin, empagliflozin, canagliflozin, diabetes, cardiovascular outcomes, oral glucose lowering drugs, type 2 diabetes mellitus, sglt2 inhibitors, anti-diabetic drugs

## Abstract

Optimal blood glucose control helps reduce the development of the complications of type II diabetes mellitus (T2DM). T2DM patients usually are at increased risk of cardiovascular (CV) events and mortality. Therapies and strategies to treat diabetes and its related CV outcomes still need more investigation to find the best management options for this population. Sodium-glucose cotransporter-2 (SGLT-2) inhibitors have several benefits over multiple organ systems of the human body. However, the comparative effectiveness of this drug class is still not well-established. Our review aims to assess SGLT-2 inhibitors' effects on the CV complications that occur because of uncontrolled diabetes.

A comprehensive literature search was conducted on PubMed and PubMed Central to find the relevant studies that were done from 2016 through 2020 to gather data for this review article. Those studies include reviews, randomized clinical trials, systematic reviews, and meta-analyses.

Studies used in this article found an associated decrease in CV complications and mortality in patients with T2DM who received treatment with SGLT-2 inhibitors compared to the placebo group. These drugs have shown significant efficacy and safety outcomes in diabetic patients with heart disease, as they are glycosuric and diuretics, both of which are characteristics that could provide benefits to this population.

SGLT-2 inhibitors appear to reduce the risk of cardiovascular events and mortality, suggesting that the benefits of these drugs seen in people with diabetes may apply to a broad population in the real world. We recommend further studies should confirm the immense clinical benefits with SGLT-2 inhibitors in patients with T2DM.

## Introduction and background

Diabetes mellitus is a group of diseases that affects multiple organs of the human body in various ways. Type II diabetes mellitus (T2DM) had a worldwide prevalence of 10% in the general population and affected more than 415 million adults in 2013, and this number was projected to increase to 592 million by 2035. T2DM currently affects over 350 million patients globally, and cardiovascular disease (CVD) is a severe complication of T2DM, i.e., primarily associated with excess mortality and morbidity in up to 80% of patients [1].

Uncontrolled high blood sugars, insulin resistance, and elevated lipids eventually lead to long-term complications due to limited physiological adaptations and repair capacity [1]. Patients with T2DM are at a high risk of developing macrovascular events. Its treatment needs a multifactorial approach, as it results in the reduction of macrovascular complications and mortality related to type 2 diabetes (T2D) [2].

Existing antidiabetic agents (ADAs) lower blood glucose either by enhancing insulin secretion or improving insulin sensitivity. Sodium-glucose cotransporter-2 (SGLT-2) inhibitors lower blood glucose via an insulin-independent mode of action by reducing glucose renal reabsorption at the S1 segment of proximal tubules in the kidney [3]. SGLT-2 inhibitors represent a unique class of glucose-lowering therapies that have multisystem health benefits. The SGLT-2 inhibitors class represents a critical new therapeutic approach for preventing heart failure in at-risk patients with T2DM and is actively being studied for use in treating patients with heart failure, with or without T2DM [4].

SGLT-2 inhibitors can decrease hyperglycemia and visceral fat, components of the metabolic syndrome, significantly associated with CVD progression [5]. There are various mechanisms through which SGLT-2 inhibitors can be beneficial for treatment purposes. It also blocks SGLT-2 receptors, which, under normal physiologic circumstances, promote the reabsorption of glucose from the glomerular filtrate back into the blood. SGLT-2 inhibitors lead to urinary loss of glucose, glycosuria, which aids in controlling blood sugar levels and overall body weight. Water tends to follow this excess glucose in urine in a process known as osmotic diuresis. This fluid loss contributes to a reduction in blood pressure levels [3,6]. SGLT-2 inhibitors inhibit the function of sodium/hydrogen exchanger 1 (NHE1) in cardiac muscle cells, which results in decreased levels of sodium and calcium in the cardiac cell’s cytoplasm and increased calcium levels within a cell’s mitochondria. Overall, there is an ultimate decrease in the sodium content within the cardiac muscle cells. These drugs also block NHE3 in proximal renal tubules and inhibit the signaling mechanism from renal tubules to the glomerulus via the macula densa in the distal convoluted tubule. The macula densa usually serves to sense the sodium chloride (NaCl) concentration in tubular fluid and uses it as an indicator of glomerular filtration rate (GFR) or tubuloglomerular feedback. Due to this inhibition of NHE3, NaCl levels in tubular fluids are high, which ultimately results in the inhibition of NaCl reabsorption in the proximal tubule. Increased NaCl loss in urine results in accompanying fluid loss or natriuresis [7]. All the effects eventually provide benefits to patients with T2DM and lead to improved ventricular volume load, cardiac muscle cell function, and diastolic function of the left cardiac ventricle, as left ventricular diastolic dysfunction is a usual pathologic finding in T2DM patients with heart failure [3].

Recently, multiple studies evaluated the effects of SGLT-2 inhibitors on T2DM. This review aims to broadly summarize the reports of meta-analyses, reviews, randomized clinical trials, and systematic review studies to evaluate the antidiabetic and cardioprotective benefits of SGLT-2 inhibitors.

## Review

Method 

We referenced the PubMed online database throughout this review. We searched scientific data and articles from 2016 up to 2020. Before the application of inclusion criteria, a total of 3,762 papers were found. Once inclusion criteria were applied, 986 scientific papers sufficed the relevancy of this topic, which includes: (1) English language; (2) human studies; (3) T2DM patients with at risk of or with pre-existing heart disease; and (4) major adverse cardiovascular events (MACE). Of those 986 papers, we used only four articles. We included various studies in our review, including scientific assessments, randomized clinical trials, systematic reviews, and meta-analyses. All the data met the criteria for Preferred Reporting Items for Systematic Reviews and Meta-Analyses (PRISMA) and are peer-reviewed.

Results

We included the results of the seven studies in this review. All seven studies suggested a decreased risk of cardiovascular complications and mortality in T2DM patients treated with SGLT-2 inhibitors versus a placebo. We used tables to provide a quick review, followed by describing all the studies' results. 

A randomized clinical trial, EMPA-REG OUTCOME® (Empagliflozin Cardiovascular Outcome Event Trial), was focused on a mixed Asian population and used to evaluate empagliflozin's benefits in patients with T2DM [8]. They randomized patients into two groups (the treatment group, which received empagliflozin 10 mg and empagliflozin 25 mg, and the placebo-controlled group once daily). The sample size of this study was 7,020. Of these 7,020 patients, 1,517 (21.6%) participants were of Asian descent - Hong Kong, India, Indonesia, Japan, Korea, Malaysia, Philippines, Singapore, Sri Lanka, Taiwan, and Thailand. Patients were initially categorized based on their race to observe any geographic differences in the drug's effects. The researchers analyzed all the participants for four-point MACE, three-point MACE, glycemic control, body mass index (BMI) control, blood pressure control, heart rate control, serum total lipids control, serum uric acid, and estimated GFR (eGFR) by using Cox proportional hazards models [8]. They also considered the assessment of the effects based on the average age and gender of the participants. Patients were also being assessed simultaneously for any adverse effects throughout the course of the study. All the results of empagliflozin vs. placebo are similar in the Asian population in T2DM with existing cardiovascular death as compared to the overall population, including cardiovascular outcomes, glycemic control, and all-cause mortality [8].

A meta-analysis was conducted in the United States to observe the effects and safety profile of empagliflozin in T2DM patients who are at risk of cardiovascular complications [9]. This meta-analysis comprised combined data from eight placebo-controlled trials. The sample size of this study was 11,292 participants. Of those, 3,835 patients received a placebo, and 7,457 patients received 10 mg and 25 mg once-daily doses of empagliflozin [9]. They set 4-point and 3-point MACE as primary and secondary study endpoints, respectively. This study calculated the risk estimates by Cox regression analysis adjusted according to this study and both the treatment and placebo-controlled groups. Researchers also calculated hazard ratios, 95% confidence intervals, and 4-point MACE in both the placebo and treatment groups. Other calculated descriptive statistics values in this analysis also provided an estimate of time to cardiovascular death and all-cause mortality after treatment with empagliflozin and placebo. This meta-analysis suggested a reduction in risk related to 4-point MACE/primary endpoint (composite of cardiovascular death, non-fatal MI (excluding silent MI), non-fatal stroke and hospitalization for unstable angina), and 3-point MACE/secondary endpoint (composite of CV death, non-fatal MI, and non-fatal stroke) in the treatment group as compared to the placebo group [9].

A group of researchers performed a large multinational study, "The CVD-Real study" (Comparative Effectiveness of Cardiovascular Outcomes in New Users of SGLT-2 Inhibitors) with a sample size of 309,056 (154,528 patients per treatment group) individuals to compare the effectiveness of ADAs on cardiovascular outcomes in new users of SGLT-2 inhibitors versus other oral glucose-lowering drugs (oGLDs) [10]. The data based on the patient's health records were collected across six countries and used as a research information resource. The collected information was then analyzed, followed by comparison after matching both treatment groups based on baseline characteristics and countries. All data were kept unidentified. This multinational, randomized clinical trial study had shown a decreased rate of hospitalizations for heart failure and all-cause death in patients with the use of SGLT-2 inhibitors as compared to oGLDs or insulin [10]. This study calculated hazard ratios and odds ratios. At the end of the analysis, it was concluded that SGLT-2 inhibitor use was associated with an overall lower risk of hospitalizations for heart failure and death, suggesting that the benefits seen with empagliflozin may apply to a general population of patients with T2DM in the real-world [10].

A multinational study, “DECLARE-TIMI 58” (Dapagliflozin Effect on Cardiovascular Events-Thrombolysis in Myocardial Infarction 58), randomized 17,160 patients with T2DM and either established atherosclerotic cardiovascular disease (n=6,974) or multi-risk factors (n=10,186) to dapagliflozin versus placebo [11]. The researchers prespecified two primary endpoints, 4-point MACE and 3-point MACE. They observed effects specifically in patients with a history of a previous MI (n=3,584). The treatment group received 10 mg of dapagliflozin once daily. This study calculated the hazard ratios and 95% confidence intervals by using the Cox proportional hazard model. This study also calculated the event rates, relative risks, and absolute risk reductions in patients with a history of prior MI vs. no prior MI history. This study found higher event rates in T2DM patients with a history of a previous MI than patients with no history of MI for MACE (17.8% versus 7.1% with events) [11]. According to this study, patients with T2DM and a previous MI history were at increased risk of hospitalizations for heart failure and cardiovascular-related mortalities. This study concluded that dapagliflozin showed a significant decrease in the risk of MACEs, cardiovascular deaths, and hospitalizations for heart failure [11]. No significant reductions were observed in patients with atherosclerotic cardiovascular disease without a history of MI or patients solely with multiple risk factors. 

A 2010 to 2019 meta-analytic review conducted in China included 42 randomized clinical trials, with 61,076 participants enrolled based on its inclusion criteria [1]. This study investigated SGLT-2 inhibitors' effects, including canagliflozin, dapagliflozin, and empagliflozin, on established cardiovascular death and mortality in patients with T2DM. In this study, researchers randomized participants into two groups after matching baseline characteristics [1]. The treatment group received SGLT-2 inhibitors as monotherapy or add-on therapy to standard care. In contrast, the placebo group received non-SGLT-2 ADAs. This study calculated odds ratios with 95% confidence intervals to evaluate the effect size of data. The results suggested that SGLT-2 inhibitors' add-on therapy as compared with other ADAs had shown a reduction in the risk of cardiovascular outcomes (e.g., MACE, MI, all-cause mortality) [1]. SGLT-2 inhibitor monotherapy showed no effect on these events. Besides, this drug class had no impact on stroke risk reduction either as mono- or add-on therapy. This study concluded that SGLT-2 inhibitors add-on therapy was associated with a decrease in MACE, cardiovascular mortality, and all-cause mortality as compared to the non-SGLT-2 inhibitors placebo group, thus providing reliable analysis of the effects of these drugs on cardiovascular events [1].

Another meta-analysis was conducted in China to investigate the benefits and safety profile of SGLT-2 inhibitors vs. oGLDs in T2DM patients with established cardiovascular diseases [12]. The researchers searched data from the year 1980-2019 and eventually used 91 randomized clinical trials based on 171,253 participants, and out of these, 4163 reported heart failure incidences. They compared the efficacy of SGLT-2 inhibitors with that of oGLDs, including dipeptidyl peptidase-4 inhibitors (DPP4i) and glucagon-like peptide-1 receptor agonists (GLP1a). The study performed pairwise direct and indirect comparisons among all the ADA classes and analyzed the drug's effects in patients. The researchers calculated odds ratios and 95% confidence intervals and then they used a rankogram for ranking the risks associated with heart failure and pairwise comparisons among drugs to calculate the surface under the cumulative ranking curves (SUCRA) [12]. The results of this study had shown that SGLT-2 inhibitors were significantly superior to other ADAs in terms of cardiovascular outcomes, as they had established a significant reduction in heart-related risks when used in patients with a high risk of heart failure [12]. The SUCRA ranking model showed that SGLT-2 inhibitors had the lowest risk of heart failure (93.4%) and thiazolidinediones (TZD) had the most significant chance of heart failure (4.3%). This study concluded that SGLT-2 inhibitors were the most recommended option as compared to other ADAs; SGLT-2 inhibitors and metformin had shown superiority in terms of cardiovascular benefits over DPP4 inhibitors and GLP1a [12]. However, the comparison of different drug combinations did not yield any significant difference.

A network meta-analysis performed in China included various studies to compare the effectiveness of newer anti-diabetic drugs vs. a placebo [6]. One of the comparisons was SGLT-2 inhibitors vs. placebo, which included EMPE-REG OUTCOME (empagliflozin vs. placebo), CANVAS (canagliflozin vs. placebo), CANVAS-R (canagliflozin vs. placebo), DECARE-TIMI 58 (dapagliflozin vs. placebo), and CREDENCE (canagliflozin vs. placebo). This study concluded that SGLT-2 inhibitors show clear superiority in reducing cardiovascular deaths, all-cause deaths, hospitalization for heart failure (HF), and renal events among new ADAs as compared to other oGLDs. This study aimed at the perspective that SGLT-2 inhibitors should now be considered the preferred treatment for T2DM [6].

Table 1 organizes the studies used in this review, which met inclusion criteria, and the conclusions drawn from them.

**Table 1:** Description of the selected studies that met the inclusion criteria for this review SGLT-2: sodium-glucose cotransporter-2; oGLDs: oral glucose-lowering drugs (e.g. metformin, sulfonylureas, dipeptidyl peptidase-4 inhibitors); thiazolidinediones: glucagon-like peptide-1 receptor agonists; MACE (major adverse cardiovascular events): defined as the composite of nonfatal myocardial infarction, nonfatal stroke, and cardiovascular mortality); T2DM: type-2 diabetes mellitus; HHF: hospitalization for heart failure; MI: myocardial infarction; ADAs: antidiabetic drug classes

Study	Study Location	Study Period	Sample Size	Conclusion of the Study
Kaku et al. [8] Randomized controlled trial	Asia (Hong Kong, India, Indonesia, Japan, Korea, Malaysia, Philippines, Singapore, Sri Lanka, Taiwan, Thailand).	2015	7020	Asian patients treated with empagliflozin had shown a reduction in the risk of cardiovascular outcomes and mortality.
Salsali et al. [9] Randomized controlled trial	USA	2015	11,292	Empagliflozin was associated with a risk reduction of MACE in patients with any cardiovascular risk factors as compared to placebo.
Kosiborod et al. [10] Randomized controlled trial	Multinational Study (United States, Norway, Denmark, Sweden, Germany, and the UK)	2013-2017	309,056	Treatment with SGLT-2inhibiotrs versus oGLDs was associated with lower rates of HHF and death.
Furtado et al. [11] Randomized controlled trial	Multinational study (USA, Canada, Mexico, Argentina, Brazil, Hungary, Australia, Belgium, Bulgaria, China, Hongkong, Czechia, France, Germany, India, Israel, Italy, Japan, Korea, Netherlands, Philippines, Poland, Romania, Russia, Slovakia, Spain, South Africa, Sweden, Taiwan, Thailand, Turkey, Ukraine, UK, Vietnam)	2013-2018	17,160	Dapagliflozin appears to robustly reduce the risk of MACE and cardiovascular death/HHF in patients with T2DM and previous MI.
Zou et al. [1] Systematic review and meta-analysis	China	2010-2019	61,076	This meta-analysis evaluated the effects of SGLT-2 inhibitors on cardiovascular outcomes in patients with T2DM and the results demonstrated that patients treated with SGLT-2 inhibitors, especially as add-on therapy, experienced significant cardioprotective effects and a potentially favorable outcome for all-cause mortality.
Yang et al. [12] A meta-analysis	China	1980-2019	171,253	Out of all classes of anti-diabetic drugs used in this study, SGLT-2 inhibitors were most beneficial in patients with heart failure.
Fei et al. [6] A network meta-analysis	China	2019	121,047	SGLT-2 inhibitors show clear priority in reducing cardiovascular and all-cause mortalities, HHF, and renal events among new ADAs.

Table 2 organizes the various studies used in a meta-analysis in China by Fei et al. [6] to assess multiple SGLT-2 inhibitors class drugs' efficacy and safety.

**Table 2:** SGLT-2 inhibitors vs placebo Description of studies used in the meta-analysis, done in China, which includes various studies to compare the effectiveness of newer anti-diabetic drugs vs placebo. This table demonstrates the hazard ratios of MACE and all-cause mortality in these trials. MACE: major adverse cardiovascular events; CI: confidence interval; HR: hazard ratio; EMPA-REG OUTCOME trial: Empagliflozin Cardiovascular Outcome Event Trial; DECLARE-TIMI 58: Dapagliflozin Effect on Cardiovascular Events-Thrombolysis in Myocardial Infarction; CANVAS: Canagliflozin Cardiovascular Assessment Study; CANVAS-R: CANVAS-Renal; CREDENCE: Canagliflozin and Renal Endpoints in Diabetes with Established Nephropathy Clinical Evaluation Source: [6]

Studies	Intervention	Study period	Sample size	HR (95% CI) of MACE	HR (95% CI) of all-cause mortality
EMPA-REG OUTCOME [13]	Empagliflozin vs. Placebo	2015	7,020 (4,687/2,333)	0.86 (0.74-0.99)	0.68 (0.57-0.82)
CANVAS [14]	Canagliflozin vs. Placebo	2017	4,330 (2,888/1,442)	0.88 (0.75-1.03)	0.84 (0.70-1.01)
CANVAS-R [14]	Canagliflozin vs. Placebo	2017	5,812 (2,907/2,905)	0.82 (0.66-1.01)	0.92 (0.82-1.04)
DECLARE-TIMI 58 [15]	Dapagliflozin vs. Placebo	2018	17,160 (8,582/8,578)	0.93 (0.84-1.03)	0.93 (0.82-1.04)
CREDENCE [16]	Canagliflozin vs. Placebo	2019	4,401 (2,202/2,199)	0.80 (0.67-0.95)	0.83 (0.68-1.02)

Discussion

In this review, we selected seven studies based on systematic reviews, meta-analyses, and randomized clinical trials with a sample size of 508,339 to assess SGLT-2 inhibitors' benefits in patients with cardiovascular diseases. We did an online search to find these articles on PubMed after applying the inclusion criteria. The uncontrolled blood sugar level is the primary risk factor for cardiovascular complications in diabetic patients. Several studies investigated the effects of various ADAs (e.g., different SGLT-2 inhibitors with oGLDs vs. placebos) in multiple countries to select the most effective treatment options in diabetic patients with cardiovascular complications. We have included various tables or figures throughout the discussion to provide a quick review of those studies' results.

In the EMPA-REG OUTCOME trial, the Asian population's findings were consistent with those of the overall population based on comparing the baseline characteristics of patients and considering all the differences in the levels of risk of cardiovascular outcomes [8]. Tables 3-5 discuss a review of the findings of cardiovascular outcomes in this study population. This study suggested that empagliflozin can significantly decrease cardiovascular effects and mortality when given with standard additional care. Empagliflozin has shown almost a reduction in 3-point MACE (14%) and cardiovascular death (38%). Even the adverse effects of findings in Asian participants were like those in the overall population [8].

**Table 3:** EMPA-REG OUTCOME trial: the assessment of benefits of empagliflozin in Asian patients with type-2 diabetes mellitus EMPA-REG OUTCOME trial: Empagliflozin Cardiovascular Outcome Event Trial; HHF: hospitalization for heart failure; CV: cardiovascular; MI: myocardial infarction; HR: hazard ratio; CI: confidence interval Source: [8]

	HR (95% CI) in the Asian population	HR (95% CI) in the overall population	Risk reduction with Empagliflozin treatment
CV death	0.44 (0.25–0.78)	0.62 (0.49–0.77)	38%
All-cause mortality	0.62 (0.49–0.77)	0.68 (0.57–0.82)	32%
HHF	0.70 (0.37–1.33)	0.65 (0.50–0.85)	35%
Composite of HHF or CV death	0.57 (0.36–0.89)	0.66 (0.55–0.79)	14%
Stroke risk (no difference between the two groups)	0.95 (0.55– 1.64)	1.18 (0.89–1.56)	-
MI risk (no difference between the two groups)	0.62 (0.36–1.08),	0.87 (0.70–1.09)	-

**Table 4:** EMPA-REG OUTCOME trial: cardiovascular outcomes in the study population EMPA-REG OUTCOME trial: Empagliflozin Cardiovascular Outcome Event Trial; MACE: major adverse cardiovascular events; CI: confidence interval; HR: hazard ratio Source: [8]

Cardiovascular outcomes in the study population				
3-point MACE (primary outcome)	Number of patients showed a decreased risk reduction	Risk Reduction (%)	HR (95% CI)	The P-value for treatment by race interaction
Empagliflozin group (overall population)	490/4,687 patients	10.5%	0.86 ( 0.74–0.99)	0.0872
Placebo group (overall population)	282/2,333 patients	12.1%		
Empagliflozin group (Asian population)	79/1,006 patients	7.9%	0.68 (0.48–0.95)	
Placebo group (Asian population)	58/511 patients	11.4%		
4-point MACE (secondary outcome)	Number of patients showed a decreased risk reduction	Risk Reduction (%)	HR (95% CI)	The P-value for treatment by race interaction
Empagliflozin group (Asian population)	101/1,006 patients	10.0%	0.73 (0.54–1.00) for the Asian race is consistent with the HR of the overall population that is 0.89 (0.78–1.01).	0.2988
Placebo group (Asian population)	69/511 patients	13.5%		

**Table 5:** EMPA-REG OUTCOME trial: glycemic control in Asian patients (empagliflozin vs. placebo) EMPA-REG OUTCOME trial: Empagliflozin Cardiovascular Outcome Event Trial; HbA1c: glycosylated hemoglobin A1c; CI: confidence interval; HR: Hazard ratio Source: [8]

	Adjusted mean differences in HbA1c in Asian patients receiving empagliflozin vs placebo					
	At 12 weeks		At 94 weeks		At 206 weeks	
	Empagliflozin (10mg, 25mg)	Placebo	Empagliflozin (10mg, 25mg)	Placebo	Empagliflozin (10mg, 25mg)	Placebo
Number of patients (n)	988	503	922	463	100	40
HR (95% CI) of Empagliflozin 10mg vs placebo	−0.48% (−0.57 to −0.40)		−0.44% (−0.57 to −0.30)		−0.15% (−0.46 to 0.15)	
HR (95% CI) of Empagliflozin 25mg vs placebo	−0.64% (−0.73 to −0.55)		−0.53% (−0.66 to −0.40)		−0.49% (−0.80 to −0.19)	

In a USA-based EMPA-REG OUTCOME meta-analysis, the researchers combined eight randomized clinical trials to gather the data to investigate the clinical effects of empagliflozin in T2DM patients with any risk level of cardiovascular diseases [9]. They assessed 4-point and 3-point MACE in patients with T2DM as endpoints of this study. Table 6 shows a brief review of those findings. This study also contributed to the conclusion of SGLT-2 inhibitors' beneficence, specifically empagliflozin, in terms of cardiovascular outcomes and mortality in T2DM patients regardless of risk levels (low, medium, or high cardiovascular risk) [9].

**Table 6:** Comparison of reduction of risk associated with empagliflozin treatment vs placebo in patients with type II diabetes mellitus to assess cardiovascular outcomes and mortality HR: hazard ratio; CI: confidence interval; MI: myocardial infarction; MACE: major adverse cardiovascular events; 4-point MACE (primary endpoint): composite of cardiovascular death, non-fatal MI (excluding silent MI), non-fatal stroke, and hospitalization for unstable angina; 3-point MACE (secondary endpoint): composite of cardiovascular death, non-fatal MI, and non-fatal stroke Source: [9]

	Empagliflozin	Placebo	HR (95% CI) for empagliflozin vs. placebo	A significance level of this meta-analysis
	Number of patients (%)	Number of patients (%)		
4-point MACE	635 (8.5%)	365 (9.5%)	0.86 (95% CI 0.76, 0.98)	α = 0.025 (2.5%), one-sided
3-point MACE	522 (7.0%)	307 (8.0%)	0.84 (95% CI 0.73, 0.96)	

**Figure 1:**
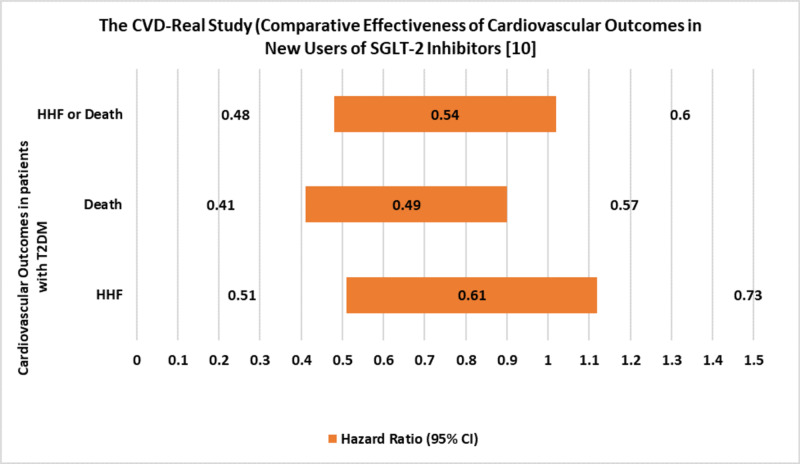
The CVD-Real Study (Comparative Effectiveness of Cardiovascular Outcomes in New Users of SGLT-2 Inhibitors) HHF: hospitalization for heart failure; CVD: cardiovascular diseases.

The “CVD-Real Study” collected and analyzed data from different countries, followed by comparing both treatment groups after matching the baseline characteristics [10]. A brief review of the results is shown in Figure 1. This study showed a 39% decreased incidence rate of hospitalizations for heart failure and all-cause death in patients treated with SGLT-2 inhibitors than those treated with oGLDs or insulin. This study also suggested that SGLT-2 inhibitors might prevent heart failure in patients at low risk of cardiovascular complications or unestablished cardiovascular disease [10]. A brief review of the findings is depicted in Table 7. Additionally, these observations were consistent across all six countries and were included even after additional adjustments in multiple categorical variables (including age, sex, geographic regions, etc.) or differences in healthcare and any particular drug use of SGLT-2 class, e.g., canagliflozin used in USA and dapagliflozin in Europe) [10].

**Table 7:** The CVD-Real Study (Comparative Effectiveness of Cardiovascular Outcomes in New Users of SGLT-2 Inhibitors) HHF: hospitalization for heart failure; CVD: cardiovascular diseases; HR: hazard ratio; CI: confidence interval Source: [10]

	HR (95% Cl)	P-value	A decrease in the Incidence rate
HHF	0.61 (0.51–0.73)	<0.001	39%
Death	0.49 (0.41–0.57)	<0.001	51%
HHF or Death (combined endpoint)	0.54 (0.48–0.60)	<0.001	46%

**Figure 2:**
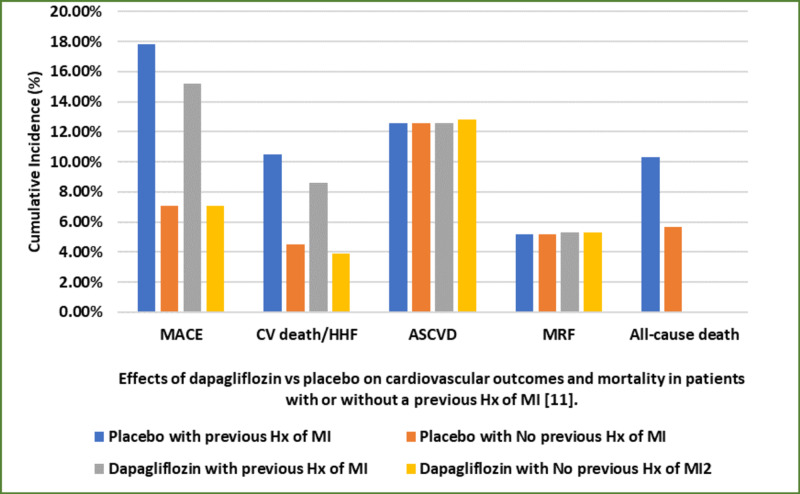
Effects of dapagliflozin vs placebo on cardiovascular outcomes and mortality in patients with or without a previous history of MI MACE: major adverse cardiovascular events; MRF: multiple risk factors; MI: myocardial infarction; ASCVD: atherosclerotic cardiovascular disease; HHF: hospitalization for heart failure

**Figure 3:**
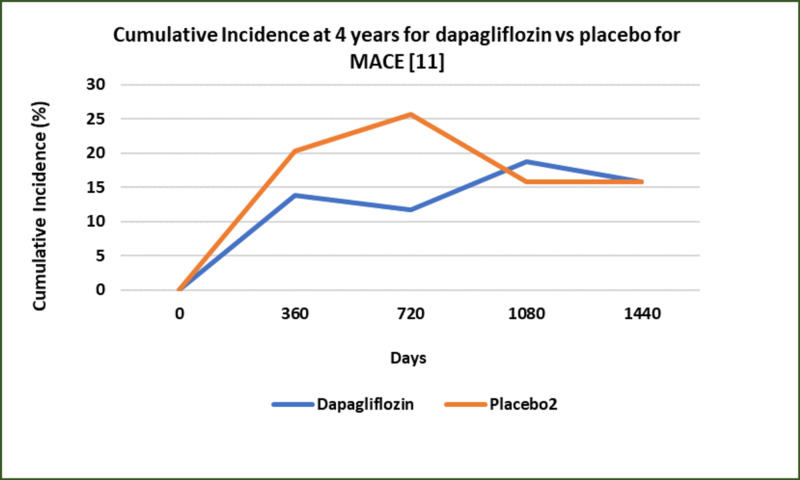
Effects of dapagliflozin vs placebo on cardiovascular outcomes and mortality in patients with or without a previous history of MI MI: myocardial infarction; MACE: major adverse cardiovascular events

DECLARE-TIMI 58, a randomized clinical trial, assessed the effects of dapagliflozin in patients with T2DM and a history of previously diagnosed atherosclerotic cardiovascular disease with or without a history of previous MI or a presence of multiple cardiovascular risk factors [11]. This study observed that dapagliflozin showed a significant decrease in both hospitalizations for heart failure and mortality in these patients with a higher relative risk and absolute risk reductions (Figure 2 and Figure 3) [11]. No significant reductions were observed in patients with atherosclerotic cardiovascular disease without a history of MI or patients solely with multiple risk factors. This study concluded that the treatment of patients with T2DM and MI history should consider SGLT-2 inhibitors [11].

A systematic review and meta-analysis based on 42 randomized clinical trials were conducted in China to evaluate SGLT-2 inhibitors' effects on cardiovascular complications and mortality in patients with T2DM [1]. In this study, researchers assessed the impact of three different SGLT-2 inhibitors, including canagliflozin, dapagliflozin, and empagliflozin, to observe and compare their effects using them as monotherapy or as add-on therapy when compared to other ADAs [1]. This study concluded that SGLT-2 inhibitors were associated with decreased risk of cardiovascular outcomes and deaths (Table 8) in patients with T2DM when compared to those treated with other ADAs [1].

**Table 8:** Description of results of the study done to evaluate SGLT-2 Inhibitors on cardiovascular outcomes and mortality in patients with type 2 diabetes MACE: major adverse cardiovascular events; MI: myocardial infarction; CI: confidence interval; OR: odds ratio Source: [1]

	OR	95% CI	P-value
MACE	0.86	0.80–0.93	<0.0001
MI	0.86	0.79–0.94	<0.0001
Cardiovascular Mortality	0.74	0.67–0.81	<0.001
All-cause Mortality	0.85	0.79-0.92	<0.0001

A network meta-analysis in China assessed the pairwise comparison of different ADAs to determine their ranking of one specific cardiovascular outcome, that is, heart failure [12]. The data searched was done on studies conducted over the past 19 years (from 1980-2019) and, eventually, the researchers selected 92 randomized clinical trials after applying the inclusion criteria. This study suggested the individual benefits of drugs on heart failure, compared those drugs to each other, and analyzed the effects of those drugs in patients with a high risk of heart failure [12]. Results had shown the significant efficacy of SGLT-2 inhibitors and metformin as compared to other ADAs. This study ranked these two drugs as the safest of all others in high-risk patients. Besides, SGLT-2 inhibitors had shown even better efficacy in these patients than metformin [12]. In contrast, the results of other combinations of ADAs did not show any significant statistical changes. This study found that TZD had shown the most significant risk of heart failure concerning other ADAs used as TZD could precipitate or exacerbate heart failure in patients with an established diagnosis of congestive heart failure (CHF) [12]. This finding further strengthens the criteria of not using TZD in CHF patients. Tables 9-10 show the calculated odds ratios [12].

**Table 9:** Anti-diabetic drugs ranking by their respective probability to be the best treatment for heart failure endpoints ADAs: anti-diabetic drugs; SUCRA: surface under the cumulative ranking curves; SGLT-2 inhibitors: sodium-glucose cotransporter-2 inhibitors; MET: metformin; INS: insulin; SU: sulfonylureas; DPP4i: dipeptidyl peptidase-4 inhibitors; PLA: placebo; GLP1a: glucagon-like peptide-1 receptor agonist; TZD: thiazolidinediones Source: [12]

ADAs	SUCRA	Interpretation of statistical significance of drugs compared to SGLT-2 inhibitors (least risk to greatest risk)
SGLT-2 inhibitors	93.4% (highest SUCRA)	Lowest risk of HF
MET	75.4%	-
INS	64.4%	-
Sulfonylureas	50.8%	-
DPP4i	44.7%	-
PLA	35.1%	-
GLP1a	31.9%	-
TZD	4.3% (lowest SUCRA)	The highest risk of HF

**Table 10:** A pairwise mixed comparison of SGLT-2 inhibitors with other ADAs for heart failure An odds ratio (OR) lower than 1 indicates better safety for heart failure. SGLT-2 inhibitors: sodium-glucose cotransporter-2 inhibitors; ADAs: anti-diabetic drugs; ADINS: insulin; DPP4i: dipeptidyl peptidase-4 inhibitors; GLP1a: glucagon-like peptide-1 receptor agonist; TZD: thiazolidinediones; MET: metformin; SU: sulfonylureas; CI: confidence interval Source: [12]

Comparison of SGLT-2 inhibitors with other ADAs for heart failure		OR (95% CI)
SGLT-2 Inhibitor vs.	INS	0.75 (0.62–0.91)
	DPP4i	0.68 (0.59–0.78)
	GLP1a	0.65 (0.54–0.78)
	TZD	0.46 (0.27–0.77)
	MET	0.53 (0.29–0.95)
	SU	0.66 (0.48–0.90)
Comparison of SGLT-2 inhibitors with other ADAs used among patients with a high risk of heart failure		OR (95% CI)
SGLT-2 Inhibitor vs.	MET	0.75 (0.58,0.95)
	SU	0.23 (0.05,1.07)
	DPP4i	0.67 (0.58,0.79)
	PLA	0.27 (0.004,1.65)
	GLP1a	0.63 90.52,0.77)
	TZD	0.15 (0.03,0.84)

**Figure 4:**
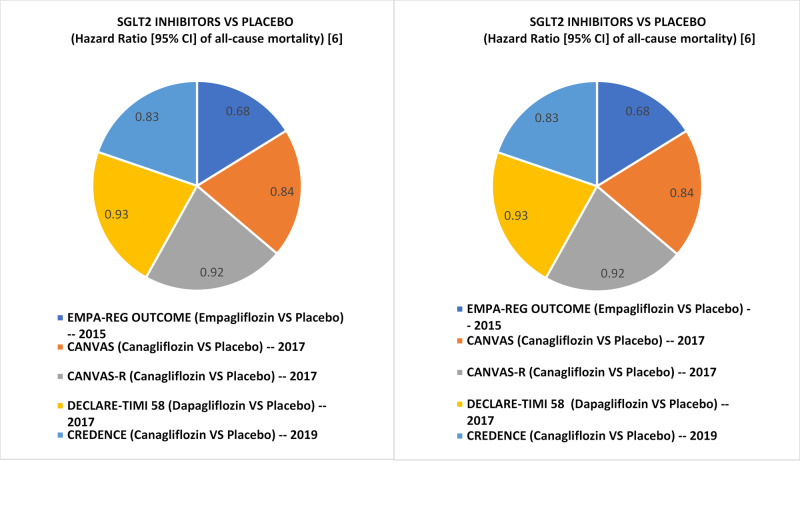
Hazard ratios of each drug trial for MACE and all-cause mortality EMPA-REG OUTCOME trial: Empagliflozin Cardiovascular Outcome Event Trial; DECLARE-TIMI 58: Dapagliflozin Effect on Cardiovascular Events-Thrombolysis in Myocardial Infarction; CANVAS: Canagliflozin Cardiovascular Assessment Study; CANVAS-R: CANVAS-Renal; CREDENCE: Canagliflozin and Renal Endpoints in Diabetes with Established Nephropathy Clinical Evaluation

Another meta-analysis combined the outcomes of 14 clinical trials conducted to evaluate the benefits of various ADA classes, including SGLT-2, glucagon-like peptide-1 receptor agonists (GLP1 RAs), and dipeptidyl peptidase-4 inhibitors (DPP4) inhibitors, on a treatment group versus a placebo-controlled group [6]. This study calculated odds ratios and 95% confidence intervals using random-effects models (Figure 4). This meta-analysis found that SGLT-2 inhibitors had shown a reduced rate of hospitalization for heart failure as compared to other ADAs and a clear superiority over others [6]. Tables 11-12 provide a quick review of these findings.

**Table 11:** A sensitivity analysis of the effect of ADAs on all-cause mortality in the comparison between SGLT-2 inhibitor vs placebo ADAs: anti-diabetic drugs; SGLT-2: sodium-glucose cotransporter-2; OR: odds ratio Source: [6]

Study	Before excluding study					After excluding study				
	OR	p-value	I^2^	Chi^2^	p-value	OR	p-value	I^2^	Chi^2^	p-value
EMPA-REG OUTCOME [13]	0.85 (0.78-0.92)	<0.0001	50%	8.07	0.09	0.89 (0.81-0.97)	0.009	0%	1.28	0.73
CANVAS [14]	0.85 (0.78-0.92)	<0.0001	50%	8.07	0.09	0.85 (0.78-0.93)	0.0003	63%	8.06	0.04
CANVAS-R [14]	0.85 (0.78-0.92)	<0.0001	50%	8.07	0.09	0.84 (0.77-0.91)	<0.0001	60%	7.57	0.06
DECLARE-TIMI 58 [15]	0.85 (0.78-0.92)	<0.0001	50%	8.07	0.09	0.79 (0.71-0.88)	<0.0001	35%	4.63	0.20
CREDENCE [16]	0.85 (0.78-0.92)	<0.0001	50%	8.07	0.09	0.85 (0.78-0.93)	0.0003	62%	7.98	0.05

**Table 12:** Hazard ratios (SGLT-2 inhibitor vs. placebo) of the outcomes evaluated in the cardiovascular outcome trials 95% CI = 95% confidence interval; HF = heart failure; HR = hazard ratio; MI = myocardial infarction; SGLT-2 = sodium-glucose cotransporter-2 Source: [6]

Studies: SGLT-2 inhibitor vs. placebo	HR (95% CI) of non-fatal MI	HR (95% CI) of nonfatal stroke	HR (95% CI) of hospitalization for HF	HR (95% CI) of composite renal outcome
EMPA-REG OUTCOME [13]	0.87 (0.70-1.09)	1.24 (0.92-1.67)	0.65 (0.50-0.85)	0.54 (0.70-0.75)
CANVAS [14]	0.85 (0.61-1.19)	0.97 (0.70-1.35)	0.56 (0.38-0.83)	0.56 (0.41-0.75)
CANVAS-R [14]	0.85 (0.69-1.05)	0.82 (0.57-1.18)	0.67 (0.52-0.87)	0.71 (0.45-1.11)
DECLARE-TIMI 58 [15]	0.89 (0.77-1.01)	1.01 (0.84-1.21)	0.73 (0.61-0.88)	0.53 (0.43-0.66)
CREDENCE [16]	Not applicable	Not applicable	0.61 (0.47-0.80)	0.66 (0.53-0.81)

After reviewing the information provided in these studies, we interpreted that the use of SGLT-2 inhibitors is associated with an overall decrease in the rate of cardiovascular outcomes, hospitalizations for heart failure, and associated cardiovascular deaths when used as single-drug therapy or add-on therapy with oGLDs as compared to other anti-diabetic drugs used alone in a real-world scenario. SGLT-2 inhibitors show remarkable benefits in T2DM patients with an existing atherosclerotic cardiovascular disease and a previous MI history. There was no improvement witnessed in patients without a history of a prior MI or only risk factors.

The analysis of our review supports the concept of the beneficence of SGLT-2 inhibitors in T2DM patients, as these drugs had shown a more remarkable improvement of glycemic index, weight control, blood pressure reduction, and, most of all, MACE, hospitalizations for heart failure, and cardiovascular mortalities. Our study included randomized controlled trials, systematic reviews, and meta-analyses, which comprised larger sample sizes of participants selected worldwide.

Our interpretation is consistent with the conclusions of the studies used in this review. Therefore, in conclusion, we assume that SGLT-2 inhibitors should be considered primary first-line treatment therapy over other antidiabetic drugs, as these drugs had shown a potential role in T2DM management with important clinical implications.

Limitations of the study

We conducted this review to find out the exact proven information regarding our interest concept, but our study had faced a few limitations as described below:

1) We preferred the study period based on the past five years (2016-2020) to determine the most recent studies done to assess this concept in patients with T2DM.

2) We tried to use articles with significant sample sizes, but we still are unsure how accurate were the case follow-ups and how much information or factors were missed out at the time the research was done.

3) We selected studies conducted in the English language only. We did not include any other languages, which might have caused us to miss some more valuable studies that could strengthen our review.

4) Several studies used for our review might have failed to mention prior patient comorbidities that may have impacted or skewed overall outcomes.

## Conclusions

Our review aimed to evaluate the effects of SGLT-2 inhibitors on cardiovascular-related comorbidities in T2D. Based on the studies conducted and the results yielded, SGLT-2 inhibitors showed promising and beneficial outcomes as compared to traditional antidiabetic drugs or placebos. Our review determined that the use of SGLT-2 inhibitors in patients with T2DM accompanied by cardiovascular complications is associated with a lower risk of hospitalizations for heart failure and mortality. It provides a wide range of benefits, including glycemic index and blood pressure regulations and ventricular volume load improvement. Future analytical studies of various drugs within the SGLT-2 inhibitor class and their side-effect profiles will help provide a more accurate statement on this drug class’s long-term benefits and fulfill the gap in the knowledge of pharmacologic mechanisms related to its effects on patients. We recommend that more research be conducted on humans to support the evidence found in the studies mentioned above. This review provides a quick overview of how the use of SGLT-2 inhibitors can be beneficial in a general population with T2DM and will significantly impact the future of medicine and research.

## References

[1] Zou C, Liu X, Sang Y, Wang B, Liang J (2019). Effects of SGLT2 inhibitors on cardiovascular outcomes and mortality in type 2 diabetes: a meta-analysis. Medicine (Baltimore).

[2] Rahelić D, Javor E, Lucijanić T, Skelin M (2017). Effects of antidiabetic drugs on the incidence of macrovascular complications and mortality in type 2 diabetes mellitus: a new perspective on sodium-glucose co-transporter 2 inhibitors. Ann Med.

[3] Tentolouris A, Vlachakis P, Tzeravini E, Eleftheriadou I, Tentolouris N (2019). SGLT2 inhibitors: a review of their antidiabetic and cardioprotective effects. Int J Environ Res Public Health.

[4] Vaduganathan M, Januzzi J Jr (2019). Preventing and treating heart failure with sodium-glucose co-transporter 2 inhibitors. Am J Cardiol.

[5] Fioretto P, Avogaro A (2017). Dapagliflozin: potential beneficial effects in the prevention and treatment of renal and cardiovascular complications in patients with type 2 diabetes. Expert Opin Pharmacother.

[6] Fei Y, Tsoi M, Cheung B (2019). Cardiovascular outcomes in trials of new antidiabetic drug classes: a network meta-analysis. Cardiovasc Diabetol.

[7] Santos D, Polidoro J, Borges-Júnior F, Girardi A (2020). Cardioprotection conferred by sodium glucose cotransporter 2 inhibitors: a renal proximal tubule perspective. Am J Physiol Cell Physio.

[8] Kaku K, Lee J, Mattheus M, Kaspers S, George J, Woerle H-J (2017). Empagliflozin and cardiovascular outcomes in Asian patients with type 2 diabetes and established cardiovascular disease. Circ J.

[9] Salsali A, Kim G, Woerle H-J, Broedl U-C, Hantel S (2016). Cardiovascular safety of empagliflozin in patients with type 2 diabetes: a meta-analysis of data from randomized placebo-controlled trials. Diabetes Obes Metab.

[10] Kosiborod M, Lam C, Kohsaka S (2018). Cardiovascular events associated with SGLT-2 inhibitors versus other glucose-lowering drugs: the CVD-REAL 2. J Am Coll Cardiol.

[11] Furtado R, Bonaca M, Raz I (2019). Dapagliflozin and cardiovascular outcomes in patients with type 2 diabetes mellitus and previous myocardial infarction. Subanalysis from the DECLARE-TIMI 58 Trial. Circulation.

[12] Yang D-Y, He X, Liang H-W (2019). Comparative outcomes of heart failure among existent classes of anti-diabetic agents: a network meta-analysis of 171,253 participants from 91 randomized controlled trials. Cardiovasc Diabetol.

[13] Zinman B, Wanner C, Lachin J (2015). Empagliflozin, cardiovascular outcomes, and mortality in type 2 diabetes. N Engl J Med.

[14] Neal B, Perkovic V, Mahaffey K (2017). Canagliflozin and cardiovascular and renal events in type 2 diabetes. N Engl J Med.

[15] Wiviott D, Raz I, Bonaca M (2019). Dapagliflozin and cardiovascular outcomes in type 2 diabetes. N Engl J Med.

[16] Mahaffey W, Jardine M, Bompoint S (2019). Canagliflozin and cardiovascular and renal outcomes in type 2 diabetes mellitus and chronic kidney disease in primary and secondary cardiovascular prevention groups. Subanalysis from the DECLARE-TIMI 58 Trial. Circulation.

